# Syntax Error: Variations in the Verbiage of Prescription Labels for Pediatric Liquid Medications

**DOI:** 10.7759/cureus.56039

**Published:** 2024-03-12

**Authors:** Austin J Bordelon, Paige Wilson, Bailey Book, Carrie Baker, Bryan J Donald

**Affiliations:** 1 Pediatrics, Edward Via College of Osteopathic Medicine, Monroe, USA; 2 College of Pharmacy, University of Louisiana Monroe, Monroe, USA

**Keywords:** prescription writing, liquid medication, knowledge among pharmacists, medication injury, pediatrics

## Abstract

Background

Pharmacists can modify prescriptions from prescribers for clarity and patient understanding, provided the confines of the original order are met, yet the verbiage used by pharmacists is not standardized. Prescription directions for children, especially children eight years old and younger, should be written with the verb “give” instead of “take” as their parents or caregivers are expected to administer them. Errors in prescribing, dispensing, and administering medication comprise a significant portion of preventable medical errors in children. To intervene and assist pharmacies, we must first identify and characterize the problem. This study aimed to determine if there is a relationship between prescribers and pharmacists using the verb “give” or “take” when prescribing and printing prescription labels for pediatric liquid medications. In addition, it aimed to determine if there is a relationship between chain pharmacies and independent pharmacies using the verb “give” or “take” when printing labels for pediatric liquid medications.

Methodology

The participants in this study were caregivers of children eight years old and younger who had been prescribed a new liquid medication. We recruited prescribers in North Louisiana to serve as a referral base for the study. Caregivers were referred to the study by prescribers. A rubric was created to investigate the text of prescription labels. Fisher’s exact test was used to determine the relationship between verb choice and prescribers and pharmacists, as well as the relationship between verb choice and chain pharmacies and independent pharmacies.

Results

A total of 11 (26.83%) prescriber texts used the verb “give,” while 12 (29.27%) prescriber texts used the verb “take.” Overall, 18 (43.90%) prescriber texts did not use a verb at all. Of these 18 prescriber texts that did not include a verb, 14 prescription labels used the verb “give,” and four used the verb “take.” In total, 10 (23.81%) chain pharmacy prescription labels used the verb “give,” and 10 (23.81%) chain pharmacy prescription labels used the verb “take.” The two-tailed p-value of Fisher’s exact test comparing verb choice between prescribers and pharmacists equaled 0.0001. A total of 19 (46.34%) independent pharmacy prescription labels used the verb “give,” and two (4.88%) independent pharmacy prescription labels used the verb “take.” The two-tailed p-value of Fisher’s exact test comparing verb choice between chain pharmacies and independent pharmacies equaled 0.0063.

Conclusions

The relationship between prescriber texts and pharmacist prescription labels shows a relationship between their verb choice (p = 0.0001). The relationship between chain pharmacy and independent pharmacy prescription labels shows a relationship between their verb choice (p = 0.0063). This study has illuminated how medication orders begin before they are modified, if necessary, for the patient’s clarity and understanding. This study can be used to instruct prescribers on writing more accurate prescription instructions to prevent medical errors, and it can help pharmacists recognize potential dangers and prevent them through editing.

## Introduction

Research has been done to establish best practices and identify potential risks for dispensing liquid medications for children to ensure proper dosing and prevent adverse events. These areas of research include specific labeling [1], dosing devices [2], metric units [3,4], numeric gradations [5], the health literacy of caregivers [6,7], and other materials that should accompany a prescription [8,9] to provide the best chance for correct dosing of liquid medications. Virtually, all these best practices apply to actions taken at the pharmacy where medications are dispensed.

The National Council for Prescription Drug Programs and the Institute of Medicine of the National Academies have published recommendations for prescription labeling standards, including explicit text to describe dosage and interval, simplified language, indications, and typography [5,10]. Pharmacists are encouraged to modify prescriptions from prescribers for clarity and patient understanding, provided the confines of the original order are met [11]. However, these are only recommendations, and the verbiage used by pharmacists is not standardized. Prescription directions for children, especially children eight years old and younger, should be written with the verb “give” instead of “take” as their parents or caregivers are expected to administer them. Using the verb “give” would create a more accurate prescription instruction to prevent medical errors. Errors in prescribing, dispensing, and administering medication comprise a significant portion of preventable medical errors in children [12].

As most prescriptions are filled and dispensed at pharmacies, the pharmacy plays an invaluable role in any effort to follow best practices and prevent medication errors. To intervene and assist pharmacies, we first need to identify if a problem exists and characterize that problem. This study intended to serve as a step forward toward pharmacy performance and medication safety for children. The subjects of the study were the pharmacies and their aggregate performance where liquid medications are fulfilled, while the participants of the study were the caregivers of children eight years old and younger who had been prescribed liquid medications.

The goals of this descriptive cross-sectional study were to analyze how prescribers write orders for liquid medications in pediatric populations and review how pharmacists from chain pharmacies and independent pharmacies keep or modify these orders before printing prescription labels. In this study, we hoped to determine if there is a relationship between prescribers and pharmacists using the verb “give” or “take” when prescribing and printing prescription labels for pediatric liquid medications. We also wanted to determine if there is a relationship between chain pharmacies and independent pharmacies using the verb “give” or “take” when printing labels for pediatric liquid medications.

## Materials and methods

Ethical approval

IRB 1206 “Creating a rubric for measuring pharmacy adherence to best practices when dispensing liquid medications for children” was approved on June 27, 2022, by the University of Louisiana Monroe Institutional Review Board. IRB 1211 “Measuring adherence to best practices when dispensing liquid medication for children” was approved on September 14, 2022, by the University of Louisiana Monroe Institutional Review Board.

Study funding

This project was supported by the Louisiana Board of Regents Support Fund Research and Development Program - Research Competitiveness Subprogram (reference #20130013625). Funds from this grant were utilized for the following: caregiver stipends, prescriber stipends, extended interview stipends, online rubric hosting, patient safety supplies, source document labeling, travel, telecommunications, investigator effort, and a student intern.

Informed consent

Participants’ first exposure to the study was from their children’s prescribers. They were given an instruction sheet and assisted in setting up an enrollment/consent meeting with the research staff. The enrollment/consent meeting could be face-to-face or conducted by video conference. The informed consent form was presented to the participant during the consent meeting. The participant could read the form and have any questions answered before providing informed consent. The participants were advised that they should not unnecessarily delay the child’s medical care to consider consent and that they could not participate in the study if they went to the pharmacy to pick up their child’s medication before providing informed consent. The participant and investigator signed the informed consent form if the meeting was face-to-face. If the meeting was by video conference, the investigator signed the informed consent form and documented that verbal consent was obtained. As an additional verification of consent, the rubric contained a question asking the participant if they freely provided informed consent, and a “no answer” immediately terminated the form and prevented the participant from proceeding or re-entering it.

Participant compensation

Prescribers were awarded a $20 stipend for each caregiver they referred who completed the study. Caregivers who completed the study were awarded a $40 stipend. Caregivers were awarded an additional $20 stipend for their time and effort in participating in extended interviews.

Study population

The participants in this study were caregivers of children eight years old and younger who had been prescribed a new liquid medication. The inclusion and exclusion criteria for the study are detailed in Table 1. No inclusion or exclusion was made based on age, gender, ethnicity, sexual orientation, or origin of the caregiver or prescriber.

**Table 1:** Inclusion and exclusion criteria for the study.

Inclusion criteria	Exclusion criteria
Prescriptions for commercially available liquid medications	Prescriptions for medications that must be compounded, except pre-measured powders for constitution in the pharmacy
Prescriptions for patients aged eight years old and younger	Prescriptions issued by telephone order
Pharmacy encounters conducted face-to-face inside the pharmacy building	Prescriptions transferred between pharmacies
Prescriptions issued directly from the prescriber to the patient or to the pharmacy (prescriptions issued on printed or written forms, electronic prescriptions sent directly to the pharmacy, or faxed prescriptions sent directly to the pharmacy)	Prefilled prescription orders, including new orders for previously prescribed medications or new doses of previous prescriptions on the patient’s records
Prescriptions for medications that are new to the patient and have not been prescribed for that patient before	Pharmacy encounters other than face-to-face encounters inside the pharmacy building where the pharmacist is on duty or present
	Prescriptions for children who prescribers or research staff believe are too ill to delay the pharmacy encounter for enrollment

Recruitment

**Figure 1:**
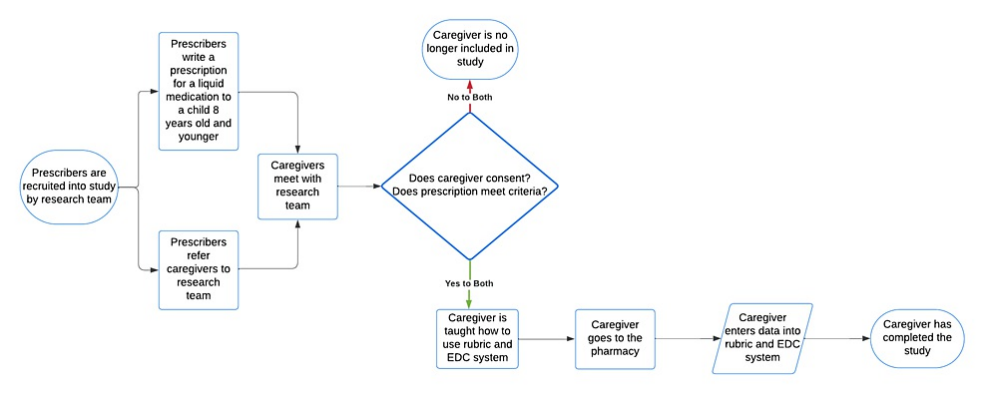
Flowchart describing how a participant entered and completed the study. EDC: electronic data capture

We recruited prescribers in North Louisiana to serve as a referral base for the study. We focused on pediatricians or prescribers who see mostly children in their regular practice. We set up meetings with prescribers to discuss the study, ask them to refer caregivers to us, and provide them with an introduction letter. Caregivers were referred to the study by prescribers, ideally at the same time a prescription for a liquid medication was issued for the caregiver’s child. Prescribers introduced the study to the caregiver and provided a letter with instructions to enter the study. To accept the invitation to enter the study, caregivers contacted the study team per the instructions in the letter before picking up their child’s medication from the pharmacy. At the enrollment meeting, caregivers were asked to provide informed consent. They verified that the prescription met the inclusion criteria, collected caregiver contact information, and instructed the caregiver regarding the proper use of the rubric and how to complete it using the electronic data capture tool. Caregivers had to complete the rubric within three days to be included in the study. The process by which a participant entered and completed the study is depicted in Figure 1. Recruitment for the study began on August 1, 2022, and ended on February 1, 2024.

Data collection

All data collection occurred through the electronic data capture system or in face-to-face meetings with a research team member. Participant information was entered by participants and/or the research team. Rubrics were completed by the participants. Physical materials were labeled and transported to an office for the research team. Caregivers who filled out rubrics and interacted with the electronic data capture system were not limited in where they must be physically located when filling out the rubric. All face-to-face meetings between participants and the research team occurred at the ULM College of Pharmacy Bienville or Shreveport campuses or in a mutually agreed-upon public venue with appropriate space to safeguard the participant’s privacy. Recruitment sites signed an agreement. This was presented to the investigator and discussed with prescribers and site personnel, and opportunities were given to ask questions before signing.

Study tools

A rubric was created to investigate how well pharmacies adhered to best practices when dispensing liquid medications for children, including the text of their prescription labels. The rubric was created by a pre-study expert panel using the Delphi method. The Delphi panel, composed of three prescribers, three pharmacists, and one parent who was not a healthcare professional, used American Academy of Pediatrics recommendations and their own experience to determine which criteria should be used in our rubric. After the Delphi panel concluded, their recommended criteria were encapsulated into a rubric intended for participants to use during the study. We developed, coded, and published an online form containing the rubric and presented that to a subgroup of the Delphi panel who had experience with surveys and survey-based research. This subgroup made recommendations and agreed that the survey tool was sound and ready for the study.

Statistical methods

A two-tailed Fisher’s exact test with p = 0.05 was used to compare chain pharmacies and independent pharmacies using the verbs “give” and “take” on their prescription labels. One data entry from a hospital pharmacy was included as an independent pharmacy. A two-tailed Fisher’s exact test with p = 0.05 was also used to compare prescriber texts and prescription labels using the verbs “give” and “take.” Because this study associates the verb “give” with promoting the safety of pediatric prescription medications, prescriber texts without a verb were included in the “take” group for statistical analysis.

## Results

A total of 41 participants entered rubrics and completed the study. The data from these rubrics is compiled in Table 2. No participants in the study completed a debriefing meeting. Overall, 48.78% of the study participants used a chain pharmacy, 48.78% used an independent pharmacy, and 2.44% used a hospital-based pharmacy. The majority of medications prescribed to the children of the study participants were antibiotics (61%).

**Table 2:** Rubric entries (n = 41).

Pharmacy type	Medication	Medication class	Prescriber text	Label given
Chain	Fluoxetine 20 mg/5 mL	Antidepressant	Give 1.25 mls by mouth once a day	Give 1.25 mls by mouth once a day
Chain	Fluoxetine 20 mg/5 mL	Antidepressant	Take 1.25 ml by mouth once a day for anxiety	Take 1.25 ml by mouth once a day for anxiety
Independent	Sulfamethoxazole-trimethoprim 200-40 mg/5 mL	Antibiotic	10 ml by mouth twice a day for 10 days. Take all doses with yogurt or probiotics and food/milk	Give 10 ml by mouth twice daily for 10 days. Take all doses with yogurt or probiotics and food/milk
Chain	Cefprozil 250 mg/5 mL	Antibiotic	6 ml by mouth twice a day for 10 days. Give all doses with probiotics or yogurt	Shake liquid well and give 6 ml by mouth twice daily for 10 days. Give all doses with probiotics or yogurt.
Independent	Prednisolone 15 mg/5 mL	Corticosteroid	Take 2 ml every day by oral route for five days	Give two (2) ml by mouth once daily for 5 days
Chain	Cetirizine 1 mg/mL	Antihistamine	3.75 ml by mouth twice a day to control itching and scratching from eczema	Take 3.75 ml by mouth twice daily
Independent	Famotidine 40 mg/5 mL	Gastric antisecretory	0.3 ml by mouth once a day to help with reflux	Give 0.3 ml by mouth every day to help with reflux. Discard any unused medication after 30 days
Chain	Cefprozil 250 mg/5 mL	Antibiotic	3 ml by mouth twice daily for 10 days. Give all doses with probiotics or yogurt	Take 3 ml by mouth twice daily for 10 days. Discard remainder
Independent	Prednisolone 15 mg/5 mL	Corticosteroid	Take 10 ml by mouth once a day	Give 10 ml by mouth every day
Chain	Cetirizine 1 mg/mL	Antihistamine	1.5 ml by mouth every night to help with sleep. Can also use for runny nose	1.5 ml by mouth every night to help with sleep. Can also use for runny nose
Independent	Azithromycin 100 mg/5 mL	Antibiotic	Take 6 ml by mouth on day one, then 3 ml by mouth daily on day 2-5	Give 6 ml by mouth on day one, then give 3 ml by mouth daily on days 2-5
Chain	Amoxicillin 400 mg/5 mL	Antibiotic	Take 12.5ml by mouth twice a day for 10 days. Take all doses with yogurt or probiotics and food/milk.	Shake liquid well and give 12.5 mL by mouth twice daily for 10 days, with yogurt or probiotics and food/milk
Chain	Cefdinir 250 mg/5 mL	Antibiotic	Give 7.5 ml by mouth every day for 10 days. Discard remaining	Give 7.5 ml by mouth every day for 10 days. Discard the remaining
Hospital	Famotidine 40 mg/5 mL	Gastric antisecretory	Give 0.3 mL per G0tube route daily. Good for 30 days once mixed. Call for refill to complete therapy	Give 0.3 ml per G-tube route daily. Good for 30 days once mixed. Call for refill to complete therapy
Chain	Amoxicillin-clavulanate 600-42.9 mg/5 mL	Antibiotic	6 ml by mouth twice a day for 10 days. Take all doses with probiotics or yogurt and food/milk	Give 6 ml by mouth twice daily for 10 days. Take all doses with probiotics or yogurt or food/milk. Discard reminder
Independent	Furosemide 10 mg/mL	Diuretic	0.4 ml per G-tube route daily	Give 0.4 ml per G-tube once daily. Discard unused portion after 90 days and refill
Chain	Cyproheptadine 2 mg/5 mL	Antihistamine	7.5 ml by mouth every evening to help with sleep	Give 7.5 mL by mouth every evening for sleep
Chain	Amoxicillin 400 mg/5 mL	Antibiotic	Take 6 ml by mouth two times a day for 10 days with food. Discard remainder	Take 6 ml by mouth two times a day for 10 days with food. Discard remainder
Independent	Propranolol 20 mg/5 mL	Antiarrhythmic	0.5 ml per G-tube route every eight hours	Give 0.5 ml per G-tube every eight hours as directed
Independent	Gabapentin 250 mg/5 mL	Antiepileptic	0.8 mils per G-tube route two times a day	Give 0.8 ml per G-tube twice daily as directed
Independent	Amoxicillin 200 mg/5 mL	Antibiotic	Take 5 ml twice a day for 10 days	Give 10 ml by mouth twice daily for 10 days
Chain	Amoxicillin-Clavulanate 600-42.9 mg/5 mL	Antibiotic	Take 5 ml by mouth twice a day for 10 days with food and yogurt to prevent diarrhea	Take 5 ml by mouth twice a day for 10 days with food and yogurt to prevent diarrhea
Chain	Amoxicillin-Clavulanate 600-42.9 mg/5 mL	Antibiotic	5 ml by mouth twice a day for 10 days. Give all doses with yogurt or probiotics and food/milk	Give 5 mls by mouth twice a day for 10 days. Give all doses with yogurt or probiotics and food/milk
Independent	Ondansetron 4 mg/5 mL	Antiemetic	Give 2.5 ml by mouth every 12 hours as needed for nausea and/or vomiting	Give 2.5 ml by mouth every 12 hours as needed for nausea and/or vomiting
Independent	Ondansetron 4 mg/5 mL	Antiemetic	Give 2.5 ml every 12 hours as needed for nausea and/or vomiting	Give 2.5 ml every 12 hours as needed for nausea and/or vomiting
Independent	Amoxicillin 400 mg/5 mL	Antibiotic	3.75 ml by mouth twice a day for 10 days	Shake liquid and give 3.75 ml by mouth twice a day for 10 days
Chain	Cefprozil 250 mg/5 mL	Antibiotic	2.5 ml by mouth twice a day for 10 days	Give 2.5 ml by mouth twice a day for 10 days
Chain	Amoxicillin 400 mg/5 mL	Antibiotic	Take 10 ml by mouth twice a day for 10 days. Give all doses with yogurt or probiotics	Take 2 teaspoonfuls by mouth twice daily for 10 days. Give all doses with yogurt or probiotics
Independent	Cyproheptadine 2 mg/5 mL	Antihistamine	2.5 mL twice a day in the morning and before supper to help her appetite and weight gain	Give 2.5 mL by mouth twice a day in the morning and before supper to help her appetite and weight gain
Independent	Cefprozil 250 mg/5 mL	Antibiotic	3 ml by mouth twice a day for 10 days	Give 3 ml by mouth twice a day for 10 days
Chain	Azithromycin 200 mg/5 mL	Antibiotic	5 mL by mouth once a day for five doses, with yogurt or probiotics and food/milk	Take 5 ml by mouth once a day for 5 days. Give all doses with yogurt or probiotics and food/milk. Discard remainder
Chain	Famotidine 40 mg/5 mL	Gastric antisecretory	0.3 ml by mouth once a day to help with reflux	Take 0.3 ml by mouth once a day to help with reflux
Chain	Amoxicillin 400 mg/5 mL	Antibiotic	7.5 ml by mouth twice a day for 10 days. Give all doses with yogurt or probiotics and food/milk	Give 7.5 ml by mouth twice daily for q0 days. Give all doses with yogurt or probiotics and food/yogurt
Independent	Sulfamethoxazole-trimethoprim 200-40 mg/5 mL	Antibiotic	10 ml by mouth twice a day for 10 days. Give all doses with yogurt or probiotics and food/milk	Take 10 ml by mouth twice daily for 10 days with yogurt or probiotics and food/milk
Independent	Cyproheptadine 2 mg/5 mL	Antihistamine	Take 5ml by mouth twice a day	Take 5ml by mouth two a day
Chain	Azithromycin 200 mg/5 mL	Antibiotic	6 mL PO today x 1 dose, then 3 mL PO QD x 4 more days	Give 6 ml by mouth today for 1 done, then 3 ml daily for 4 more days
Chain	Sulfamethoxazole-trimethoprim 200-40 mg/5 mL	Antibiotic	10 ml by mouth twice a day for an additional 10 days. Give all doses with yogurt or probiotics and food/milk	Take 2 teaspoonsfuls (10 mL) by mouth twice daily for an additional 10 days. Give all doses with yogurt or probiotics and food/milk
Chain	Azithromycin 200 mg/5 mL	Antibiotic	8 mL PO today x 1 dose, then 4 mL PO QD x 4 more days	Give 8 mL by mouth today for 1 dose, then 4 mL daily for 4 more days
Independent	Azithromycin 200 mg/5 mL	Antibiotic	7 mL PO today x 1 dose, then 3.5 mL PO QD x 4 more days	Give 7 mL by mouth today for 1 dose, then 3.5 mL daily for 4 more days
Chain	Amoxicillin 400 mg/5 mL	Antibiotic	10 ml by mouth twice a day for 10 days	Give 10mls by mouth 2 times a day for 10 days
Independent	Amoxicillin 400 mg/5 mL	Antibiotic	10 ml by mouth twice a day for 10 days	Give 10mls by mouth 2 times a day for 10 days

Prescriber texts vs. prescription labels

**Figure 2:**
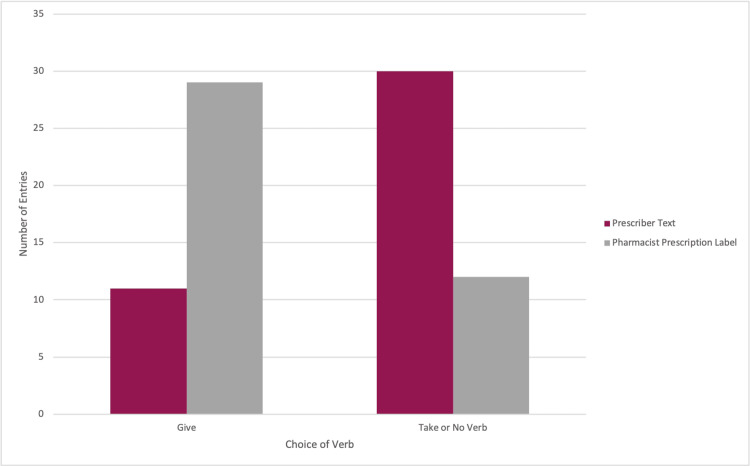
Histogram displaying the choice of the verb between prescribers and pharmacists.

Because this study associates the verb “give” with promoting the safety of pediatric prescription medications, prescriber texts without a verb were included in the “take” group for statistical analysis. A total of 11 (26.83%) prescriber texts used the verb “give,” while 12 (29.27%) prescriber texts used the verb “take.” Overall, 18 (43.90%) prescriber texts did not use a verb at all. Of these 18 prescriber texts that did not include a verb, 14 prescription labels used the verb “give,” and four used the verb “take” (Figure 2). The two-tailed p-value of Fisher’s exact test equaled 0.0001.

Chain pharmacies vs. independent pharmacies

**Figure 3:**
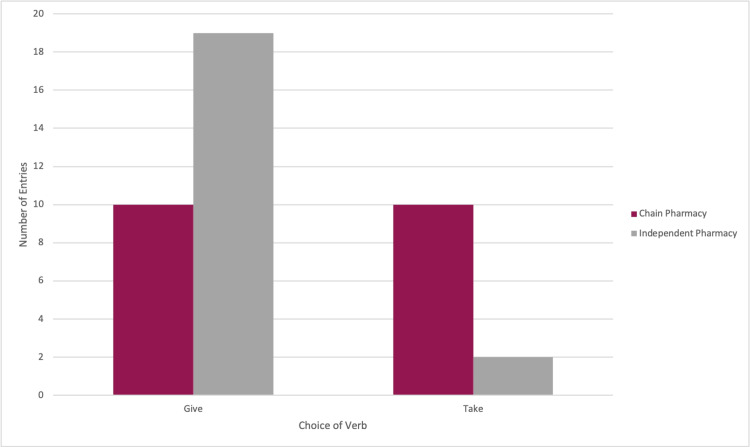
Histogram displaying the choice of the verb between chain pharmacies and independent pharmacies.

The one data set from a hospital pharmacy was included in the “independent pharmacy” group for statistical analysis. In total, 10 (23.81%) chain pharmacy prescription labels used the verb “give,” and 10 (23.81%) chain pharmacy prescription labels used the verb “take.” Overall, 19 (46.34%) independent pharmacy prescription labels used the verb “give,” and two (4.88%) independent pharmacy prescription labels used the verb “take.” Of the 18 prescription labels from prescriber texts that did not use a verb, the 14 labels that used the verb “give” came from independent pharmacies, and the four labels that used the verb “take” came from chain pharmacies (Figure 3). The two-tailed p-value of Fisher’s exact test equaled 0.0063.

## Discussion

Fisher’s exact test comparing prescriber texts and pharmacist prescription labels shows a relationship between their verb choice (p = 0.001). Prescribers were more likely to use the verb “take” or not include a verb, and pharmacists were more likely to use the verb “give” for their prescription labels. It could be inferred that pharmacists are trying to use correct verbiage on their prescription labels, demonstrated by correcting prescriber texts and inserting the correct verb when a verb was not used at all. The study did not compare hand-written prescriptions and free-text electronic prescriptions to auto-populated electronic prescriptions. This may play a role in the syntax or lack of verbs found in the prescriptions in this study. This study did not measure physician counseling, which plays a role in preventing medication injuries [13,14].

Fisher’s exact test comparing chain pharmacy and independent pharmacy prescription labels shows a relationship between their verb choice (p = 0.0063). Chain pharmacies were more likely to use the verb “take” on their prescription labels, and independent pharmacies were more likely to use “give” on their prescription labels. This relationship was also apparent when the prescriber’s text did not include a verb. This study did not include an analysis of chain or independent pharmacy operation protocols as the study participants were caregivers. This project also does not mean to serve as a repudiation of any particular pharmacist or pharmacy.

There have been no reports of accidental ingestion of a child’s medication by a caregiver on the literature review. Pham et al., however, reported on the purposeful misuse of attention-deficit/hyperactive disorder medication by parents [15].

The results of this study could be used to enhance education and recommendations for pharmacists regarding pediatric prescriptions for liquid medications. The results of this study may also impact prescribers to be conscientious when writing orders to prevent accidental injury. These results may also influence electronic medical record auditors and electronic prescription software developers to analyze how their programs populate templates for pediatric medications.

Future research could be developed to investigate if there are documented accidental medication injuries in adults taking their children’s medication due to the misuse of the verb “take.” Additional research projects may include repetitions of this project in more urban regions to see if similar trends occur, analyzing the demographics of the caregivers, and processing individual pharmacies within a particular region.

Limitations

Recruitment for this study was difficult and stagnant in its initiation, resulting in a small sample size of 41. As this study continues, we hope to see our sample size increase so that we may feel more confident in its results.

## Conclusions

This study intended to delve into the literature on the safety of pediatric liquid medication prescriptions. We presented an apparent relationship between verb choice/use and prescribers and pharmacists (p = 0.0001), as well as how this verb choice preference exists between chain pharmacies and independent pharmacies (p = 0.0063). This study has illuminated how medication orders begin before they become modified, if necessary, for the clarity and understanding of the patient. This study can help physicians and mid-level providers to become more conscientious prescribers and pharmacists to become ever more vigilant editors. This study can be used to instruct prescribers on writing more accurate prescription instructions to prevent medical errors, and it can help pharmacists recognize potential dangers and prevent them through editing.
